# Meiosis resumption in human primordial germ cells from induced pluripotent stem cells by in vitro activation and reconstruction of ovarian nests

**DOI:** 10.1186/s13287-022-03019-3

**Published:** 2022-07-26

**Authors:** Sheng Yang, Zhen Liu, Shengda Wu, Lang Zou, Yanpei Cao, Hongjia Xu, Jingfeng Huang, Qingyan Tian, Fanggui Wu, Panpan Li, Shuping Peng, Cijun Shuai

**Affiliations:** 1 The Reproduction Medical Center, The Third Affiliated Hospital of Shenzhen University, Shenzhen, 518001 Guangdong People’s Republic of China; 2 Department of Obstetrics and Gynecology, Shenzhen University General Hospital, Shenzhen, 518053 Guangdong Province People’s Republic of China; 3 NHC Key Laboratory of Carcinogenesis, The Key Laboratory of Carcinogenesis and Cancer Invasion of the Chinese Ministry of Education, Xiangya Hospital, Central South University, Changsha, 410078 People’s Republic of China; 4 Institute of Additive Manufacturing, Jiangxi University of Science and Technology, Nanchang, 330013 People’s Republic of China; 5 State Key Laboratory of High-Performance Complex Manufacturing, Central South University, Changsha, 410083 People’s Republic of China

**Keywords:** Primordial germ cells, Induced pluripotent stem cells, Meiosis, Differentiation, Oocyte, Wnt signaling pathway

## Abstract

**Background:**

The differentiation of human induced pluripotent stem cells (iPSCs) into oocytes, which involves the transformation from mitosis to meiosis, has been a hotspot of biological research for many years and represents a desirable experimental model and potential strategy for treating infertility. At present, studies have shown that most cells stagnate in the oogonium stage after differentiation into primordial germ cells (PGCs) from human iPSCs.

**Methods:**

iPSCs carrying a SYCP3-mkate2 knock-in reporter were generated by the CRISPR/Cas9 strategy to monitor meiosis status during induced differentiation from iPSCs into oocytes. These induced PGCs/oogonia were activated by small molecules from the Wnt signaling pathway and then cocultured with reconstructed human ovarian nests in vivo for further development.

**Results:**

First, human PGCs and oogonia were efficiently induced from iPSCs. Second, induced dormant PGCs resumed meiosis and then differentiated into primary oocytes through the in vitro activation of the Wnt signaling pathway. Finally, a new coculture system involving the reconstruction of ovarian nests in vitro could facilitate the differentiation of oocytes.

**Conclusions:**

Human PGCs/oogonia induced from iPSCs can be activated and used to resume meiosis by molecules of the Wnt signaling pathway. The coculture of activated PGCs and reconstruction of ovarian nests facilitated differentiation into primary oocytes and the generation of haploid human oocytes in vivo. These findings established a new strategy for germline competence in primary oocytes and provided a keystone for human gametogenesis in vitro and in vivo.

**Supplementary Information:**

The online version contains supplementary material available at 10.1186/s13287-022-03019-3.

## Introduction

Induced pluripotent stem cells (iPSCs) derived from somatic reprogramming can proliferate continuously and infinitely in vitro and have the potential for self-renewal and differentiation into various tissues and organs, including germ cells [1, 2], which provides a new method to obtain oocytes in reproductive medicine. In mammalian development, germ cells originate from primordial germ cells (PGCs). In vitro, PGCs derived from iPSCs pass through the oogonia and primary and secondary oocyte stages and finally develop into mature oocytes. The differentiation of iPSCs into mature oocytes involves the transformation from mitosis to meiosis, which has long been a frontier and hotspot of biological research [3, 4].

The generation of germ cells from mouse embryonic stem cells (ESCs) or iPSCs has been previously reported [5–12]. The reconstitution of a culture system for germ cell development using mouse pluripotent stem cells, including ESCs and iPSCs, has been established. Mouse pluripotent stem cells are in a ‘naïve’ state and can be induced into PGC-like cells (mPGCLCs) [8, 9]. Male mPGCLCs can contribute to spermatogenesis when transplanted into the testes of neonatal mice lacking endogenous spermatogenesis [8]. Female mPGCLCs can contribute to oogenesis when aggregated with embryonic ovarian somatic cells, forming isogeneic reconstituted ovaries (IROvaries) that can be transplanted under the ovarian bursa of immune-deficient mice [9]. These resultant spermatozoa or oocytes produce healthy offspring [8, 9].

In contrast, the differentiation of mature oocytes from human ESCs or iPSCs has not been achieved and has been less extensively studied [13–18]. The differentiation of human iPSCs into oocytes in vitro follows the principles of cell development in vivo. Under normal physiological conditions, not all PGCs or oogonia derived from human pluripotent precursors will continue to develop. Most of these cells exist in a dormant state. Recently, researchers have tried to differentiate human iPSCs into PGCs or oogonia and further develop them into mature oocytes by reconstituting human germ cell development using mouse embryonic ovarian somatic cells in culture. The results showed that most cells were arrested in prophase I, and only a few cells entered meiosis, indicating that in vitro differentiation from iPSCs into oocytes encountered the bottleneck of the dormant state [19, 20].

In the current study, human PGCs and oogonia were efficiently induced from iPSCs. Next, the dormant PGCs and oogonia resumed meiosis and then differentiated into primary oocytes through the in vitro activation of the Wnt signaling pathway. Finally, we developed a new coculture system by reconstructing human ovarian nests in vivo to facilitate the differentiation of oocytes. Our results also showed that the codifferentiated human activated PGCs in the reconstructed human ovarian nests resumed meiosis and differentiation into primary oocytes.

## Materials and methods

### Ethics

This study was approved by the Medical Ethics Committee of Shenzhen University. Abortion is legal in China. Fetal ovarian somatic cells were isolated from ovary tissues in aborted fetuses, which is a legal practice in China.

### Animals

All animal experiments were approved by the Ethics Committee on Laboratory Animals of Shenzhen University and were performed under ethical guidelines.

### Induction of PGCs from human iPSCs

The methods used for the generation and culture of human iPSCs and the induction of PGCs from human iPSCs were described in our previous studies [18, 19]. The main procedure was as follows. For preinduction, iPSCs were dissociated with TrypLE Express and filtered through a 50-mm cell filter, and 4 × 10^5^ cells in 12 wells were plated on vitronectin/gelatin-coated plates in N2B27 medium containing 1% KSR, 10 ng/ml bFGF (SCI), 1 ng/ml TGF-beta (PeproTech), 20 ng/ml Activin A (SCI), and 10 mM ROCK inhibitor. The medium was changed on Day 1. After 2 days of preinduction, the cells were dissociated with TrypLE and plated in ultralow-cell attachment 96-well plates (Corning, 7007) at a density of 2,000–4,000 cells/well in 200 ml of PGC medium. The PGC medium contained Glasgow’s modified Eagle medium (MEM) (GIBCO), 15% knockout serum replacement (KSR), 0.1 mM nonessential amino acids, 0.1 mM 2-mercaptoethanol, 100 U/ml penicillin–0.1 mg/ml streptomycin, 2 mM L-glutamine, 1 mM sodium pyruvate, and the following cytokines: 500 ng/ml BMP4 (R&D Systems) or BMP2 (SCI), 1 mg/ml human LIF (SCI), 100 ng/ml stem cell factor (SCF) (R&D Systems), 50 ng/ml epidermal growth factor (EGF) (R&D Systems), and 10 mM ROCK inhibitors.

### Generation of reconstituted ovarian nests in vivo

Isogeneic reconstituted ovaries (IROvaries) were generated by aggregating activated human PGCs/oogonia with fetal ovarian somatic cells of aborted 7- to 8-week-old fetuses essentially according to a procedure described for the generation of isogeneic ovaries. Briefly, the induced human PGCs/oogonia from iPSCs were dissociated into single cells, and the fetal ovarian somatic cells were collected by FACS (fluorescence-activated cell sorting). To isolate fetal ovarian somatic cells, aborted fetuses at approximately 7–8 weeks of age were used under informed consent, and the fetal tissues were dissected in chilled Dulbecco’s modified Eagle medium (DMEM) (Gibco) containing 10% fetal bovine serum (FBS) (HyClone), 2 mM GlutaMax (Gibco), 10 mM HEPES (Gibco), and 100 U/ml penicillin/streptomycin (Gibco). Fetal ovaries were identified by their appearance. The isolated ovary tissue was dissociated into single cells, and endogenous human PGCs were selected by MACS (magnetic-activated cell sorting). Human PGCs/oogonia (5000 cells/well) and human fetal ovarian somatic cells (50,000 cells/well or, when using frozen stocks, 75,000 cells/well) were plated on the wells of a Lipidure-coated U-bottom 96-well plate (Thermo Fisher Scientific) and cultured under floating conditions in GK15 + Y medium (GK15 and 10 μM ROCK inhibitor). After 2 days of floating culture, using a glass capillary, the IROvaries were transferred onto Transwell-COL membrane inserts (Corning) soaked in alpha-minimum essential medium (GIBCO) containing 10% FBS, 55 μM 2-mercaptoethanol (GIBCO), 150 nM l-ascorbic acid (SIGMA), and 100 U/ml penicillin/streptomycin. The medium was changed every three days. A mixture of in vitro-activated induced PGCs and treated human gonad cells was cultured for two days and then transplanted under the ovarian bursa of SCID mice.

### Flow cytometry

The differentiated cells were assessed using flow cytometry. The cells were first trypsin-coated into a single cell suspension, washed with 1 × PBS, and incubated with 100 μl of diluted primary antibody, followed by treatment with the respective conjugate tagged with FITC for 30 min at 4 °C (1:500). Approximately 100 μl of cell suspension containing 1 × 10^6^ cells was incubated with antibodies against CD38 with an appropriate isotype-matched control. The results were acquired and analyzed on a FACS Calibur flow cytometer (Becton Dickinson, USA). A total of 10,000 events were acquired and analyzed in each case to determine the percentage of differential expression of each cell surface marker.

### Magnetic-activated cell sorting (MACS)

Isolated ovary tissues from aborted fetuses at 7–8 weeks old were dissociated by TrypLE expression for 15 min at 37 °C and quenched with 5 times the volume of DMEM/F12 medium (GIBCO) containing 0.1% BSA fraction V. The cell suspension was passed through a nylon cell strainer and centrifuged at 1200 rpm for 3 min, and the supernatant was discarded. The cell pellet was resuspended in MACS buffer (PBS containing 0.5% BSA fraction V and 2 mM EDTA) and then incubated with anti-SSEA1 and anti-CD38 antibodies (Miltenyi Biotec) for 20 min on ice. The cell suspension was then washed with MACS buffer and centrifuged at 1200 rpm for 3 min, and the supernatant was removed. The cell pellet was resuspended in MACS buffer, and then the cell suspension was applied to an MS column (Miltenyi Biotec). The flow-through was washed with GK15 + Y and used as fetal ovarian somatic cells for the generation of human restructured ovaries.

### Real-time PCR

Total RNA was extracted and purified using an RNeasy Micro Kit (Qiagen, 74004). The main procedure was as follows. Approximately 1 ng of total RNA from each sample was used for the synthesis and amplification of cDNAs. cDNA was generated using Power SYBR Green Master Mix (Applied Biosystems) in a real-time qPCR system (Bio–Rad). The expression level of each gene was evaluated relative to the average expression levels of the 28S gene. Melting curve analysis was performed to determine the specificity of the amplified products. The relative gene expression level was calculated by subtracting the cycle threshold (Ct) value of 28S (control gene) from the Ct value of the target gene to generate the △Ct value, and the relative changes in mRNA expression were obtained using the 2^−△△Ct^ method [21]. A 95% confidence interval was accepted.

### Karyotyping

For karyotyping, a mixture of human activated PGCs and fetal somatic cells from SCID mice was cultured in PGC medium containing 0.06 µg/ml colcemid (Sigma, USA) overnight. After washing with PBS three times, the cells were incubated in medium containing 0.05% trypsin and 0.53 mM EDTA (Gibco-BRL, USA) at 37 °C for 10 min and harvested using standard procedures, followed by standard G-banding for karyotyping.

### Statistical analyses

Statistical analyses in this study were performed using SPSS version 21.0. The data are presented as the means ± standard errors of the mean (SEMs) and were analyzed by *t* tests. A *P* value of < 0.05 was considered statistically significant.

## Results

### Induction of human PGCs/oogonia from iPSCs

**Fig. 1:**
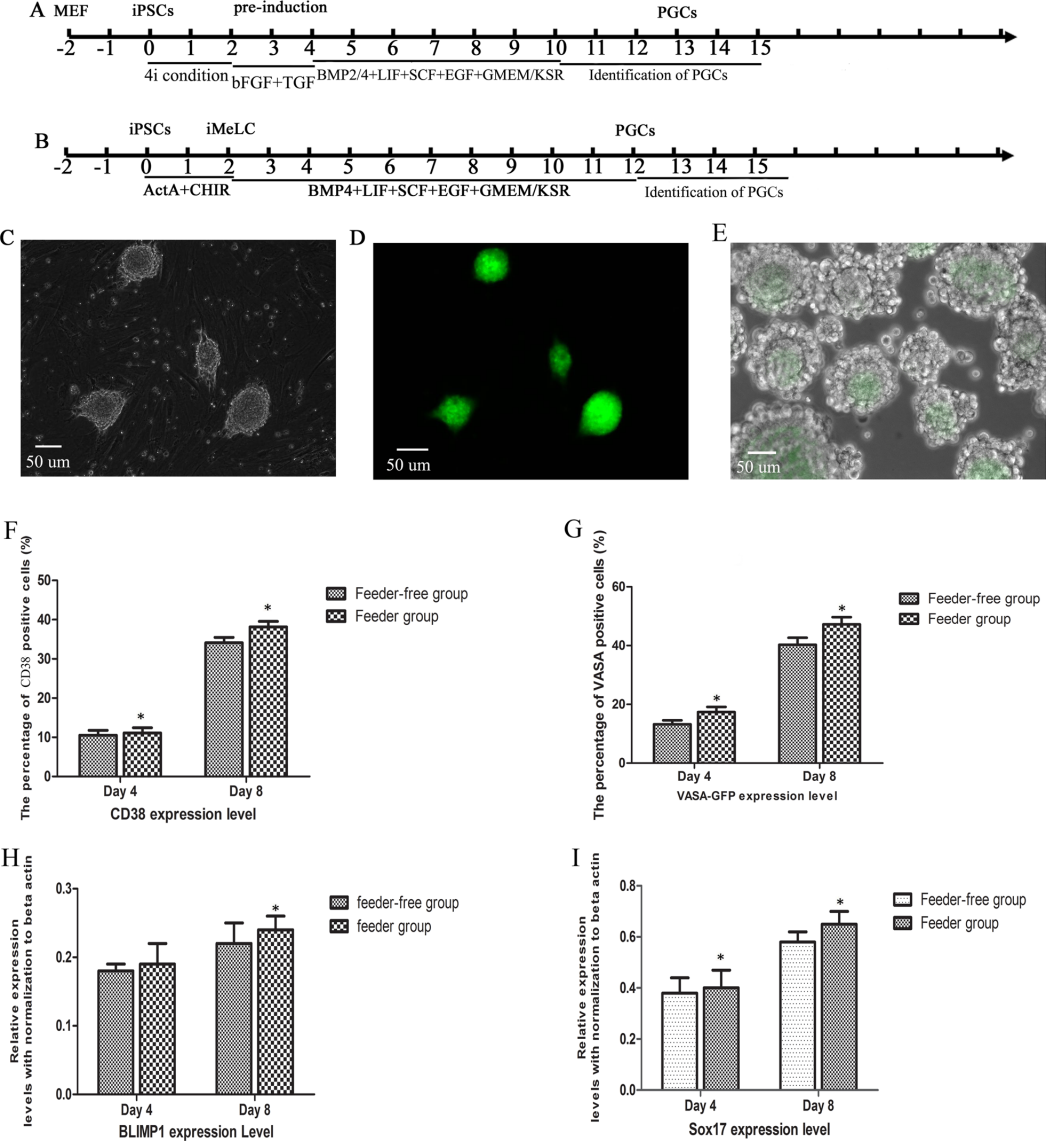
Induction of human primordial germ cells from pluripotent stem cells. **A** Timeline of the induction of human PGCs from iPSCs on feeder cells. **B** Timeline of the induction of human PGCs from iPSCs on free-feeder cells. **C** Morphology of typical VASA-GFP-iPS clones in bright field microscopy. **D** Morphology of typical VASA-GFP-iPS clones in fluorescent light. **E** VASA-GFP-iPSCs were induced into human PGCs and expressed the VASA gene. **F** Percentage of CD38-positive cells derived from iPSCs at Day 4 and Day 8. **G** Percentage of VASA-positive cells derived from iPSCs at Day 4 and Day 8. **H** Relative expression of Sox17 during PGC induction differentiation from iPSCs at Day 4 and Day 8. **I** Relative expression of BLIMP1 during PGC induction differentiation from iPSCs at Day 4 and Day 8

Human iPSCs were established as previously described [18]. These iPSCs were identified and shown to be in fully pluripotent states. In our study, human iPSCs with the XX karyotype were differentiated into primordial germ cells in two ways. Additionally, the induction efficiency of human PGCs from iPSCs was compared in two different ways. Cells produced by the first method, as described by Irie et al. [17], were named the feeder cell group (Fig. 1A). To maintain full pluripotency status, iPSCs were kept in 4i culture medium (including inhibitors of MAPK, GSK3, p38, and JNK) on feeders. These cells were preinduced with transforming growth factor-β (TGF-β) and basic fibroblast growth factor (bFGF) for 2 days, and the preinduced cells were then induced to differentiate into PGCs/oogonia by the method described by Irie et al. [17]. Cells produced by the second method, as described by Sasaki et al. [19], were called the feeder cell-free group. iPSCs cultured without feeder cells were induced into iMeLCs by ActA and CHIR99021 (CHIR) for 2 days. iMeLCs were then induced to differentiate into PGCs/oogonia (Fig. 1B). To evaluate the induction efficiency of PGCs/oogonia, we first constructed the special PGC gene reporter system VASA-GFP. The VASA-GFP reporter was transferred into human iPSCs (Fig. 1C, D). The stable VASA-GFP-iPSCs could form embryonal bodies (Fig. 1E) and were shown to be pluripotent by detecting the gene expression of the pluripotent marker and the differentiation potency, and the karyotype after transfer was normal (Additional file 1).

CD38 and VASA are both special markers of PGCs. To evaluate whether we obtained PGCs from iPSCs during induction differentiation, CD38- and VASA-GFP-positive cells were sorted by FACS. The percentages of CD38-positive cells derived from iPSCs were approximately 11.12 ± 1.31% and 38.16 ± 1.41% at Day 4 and Day 8 in the feeder group, respectively, and in the feeder-free group, the percentages were 10.51 ± 1.25% and 34.09 ± 1.38%, respectively (Fig. 1F). VASA–GFP-positive cells from iPSCs were 17.35 ± 1.75% and 47.23 ± 2.42% at Day 4 and Day 8 in the feeder group and 13.21 ± 1.35% and 40.29 ± 2.37% at Day 4 and Day 8 in the feeder-free group, respectively (Fig. 1G). Sox17 and BLIMP1 are critical determinants of the induced differentiation of human PGCs from iPSCs. The relative expression levels of Sox17 and BLIMP1 were analyzed during PGC/oogonia induction differentiation from iPSCs in the feeder group and feeder-free group. The expression levels of Sox17 and BLIMP1 both increased from Day 4 to Day 8. However, the expression level of Sox17 increased more obviously than that of BLIMP1 in the feeder group (Fig. 1H, I). The CD38, BLIMP1, and SOX17 protein levels were further identified by immunofluorescence (Additional file 2). These results showed that PGCs/oogonia were induced from iPSCs in our culture system, and a higher efficiency of PGC induction from iPSCs was achieved in the feeder group than in the feeder-free group. The iPSCs on feeder cells were then subsequently propagated and cultured for further experiments in our study.

### Reconstruction of human ovarian nests in vivo to generate human oogonia

**Fig. 2:**
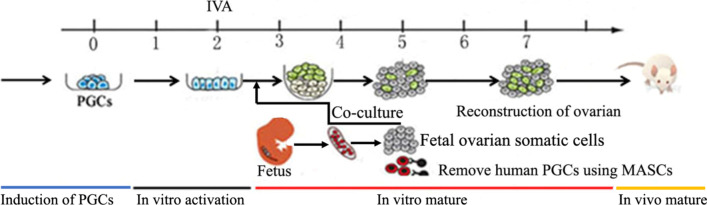
Reconstruction of the human ovarian nest in vivo for the generation of human oocytes

The gonad cells of aborted female fetuses at 7–8 weeks old were collected. Human PGCs in the gonads were removed using MACS and then cocultured with induced activated PGCs from iPSCs for two days. After in vitro activation, the mixture of induced PGCs and fetal ovarian somatic cells was transplanted into the ovarian nests of SCID mice for folliculogenesis (Fig. 2). To explore the potential of induced human PGCs for oogenesis and the efficiency of in vitro activation in the reactivation of dormant human PGCs**,** we generated reconstituted ovaries/recipient ovaries, which included a mixture of in vitro-activated induced PGCs with and treated human gonad cells. Then, the reconstituted ovaries were transplanted at day 2 of culture under the ovarian bursa of SCID mice (two reconstituted ovaries). One day after transplantation, the SCID mice received a daily intraperitoneal (i.p.) injection of follicle-stimulating hormone (FSH, 2 IU/day) to promote follicle development.

### Establishment of human iPSCs bearing the SYCP3-mkate2 reporter

**Fig. 3:**
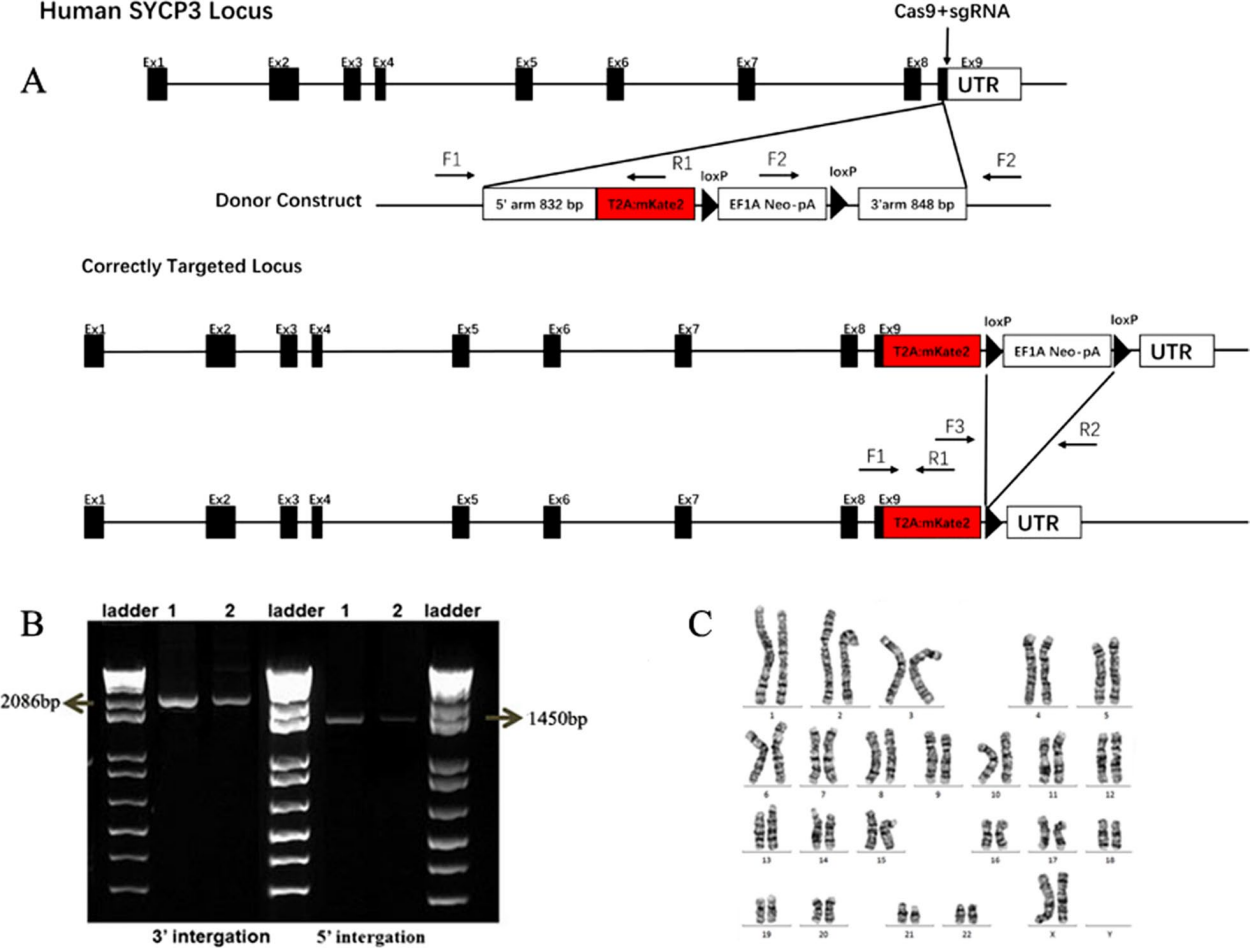
Generation of SYCP3-mkate2 reporter knock-in human iPSC lines. **A** Schematic illustration of the SYCP3 locus and the donor construct carrying T2A-mKate2 and hEF1a-Neo-pA fragments. Black boxes indicate the exons. **B** PCR screening of homologous recombinants for SYCP3-mkate2 and the removal of the selection cassettes (loxP-hEF1a-Neo-pA-loxP). Clones bearing SYCP3-mkate2 were selected for use in the subsequent studies. **C** Normal karyotype of SYCP3-mkate2 reporter knock-in human iPSC lines

Few human PGCs are activated for development into primordial follicles in the natural menstrual cycle. Most human PGCs appear to enter progressively into mitotic and meiosis prophase arrest. To explore whether the in vitro activation of dormant human PGCs causes them to resume meiosis, a SYCP3-mkate2 reporter vector was constructed. SYCP3 is expressed in human PGCs and human fetal germ cells. The SYCP3 complex is involved in synapsis, recombination and the segregation of meiotic chromosomes. The expression of SYCP3 in early human germ cells indicates the initiation of meiosis. First, we generated a SYCP3-mkate2 knock-in reporter in iPSCs by the CRISPR/Cas9 strategy described in Fig. 3A. The genome-edited iPSCs were maintained in 4i culture medium. Single cells were seeded for culture and expansion. Colonies developed after several days were used for the verification of homologous recombination by PCR (Fig. 3B), and we expanded the correctly targeted clones with normal karyotypes (Fig. 3C) for subsequent studies. Meanwhile, the derived SYCP3-mkate2 reporter knock-in human iPSCs exhibited the typical morphology of human iPSCs, expressed pluripotent markers, such as NANOG, OCT3/4, SOX2, SSEA4, and TRA-1-60, and did not express SYCP3 (Additional file 3).

### In vitro activation of dormant human PGCs by the Wnt signaling pathway overcomes meiosis phase arrest

Maturation promoting factor (MPF) is a major regulator of meiotic progression, with a complex consisting of cyclin-dependent kinase 1 (CDK1) and a regulatory subunit cyclin B1 [22–24]. Studies have confirmed that glycogen synthase kinase-3β stimulates XRINGO (an inducer of oocyte meiotic maturation) processing and further directly activates CDK1 [25], and the activation of CDK1 results in precocious meiotic resumption [26]. Based on these studies, we believe that *GSK-3β* might be an important regulator of meiotic resumption in PGCs. *GSK-3β* is an important regulator of the Wnt signaling pathway. The role and function of the Wnt signaling pathway in meiosis resumption are not clear.

**Fig. 4:**
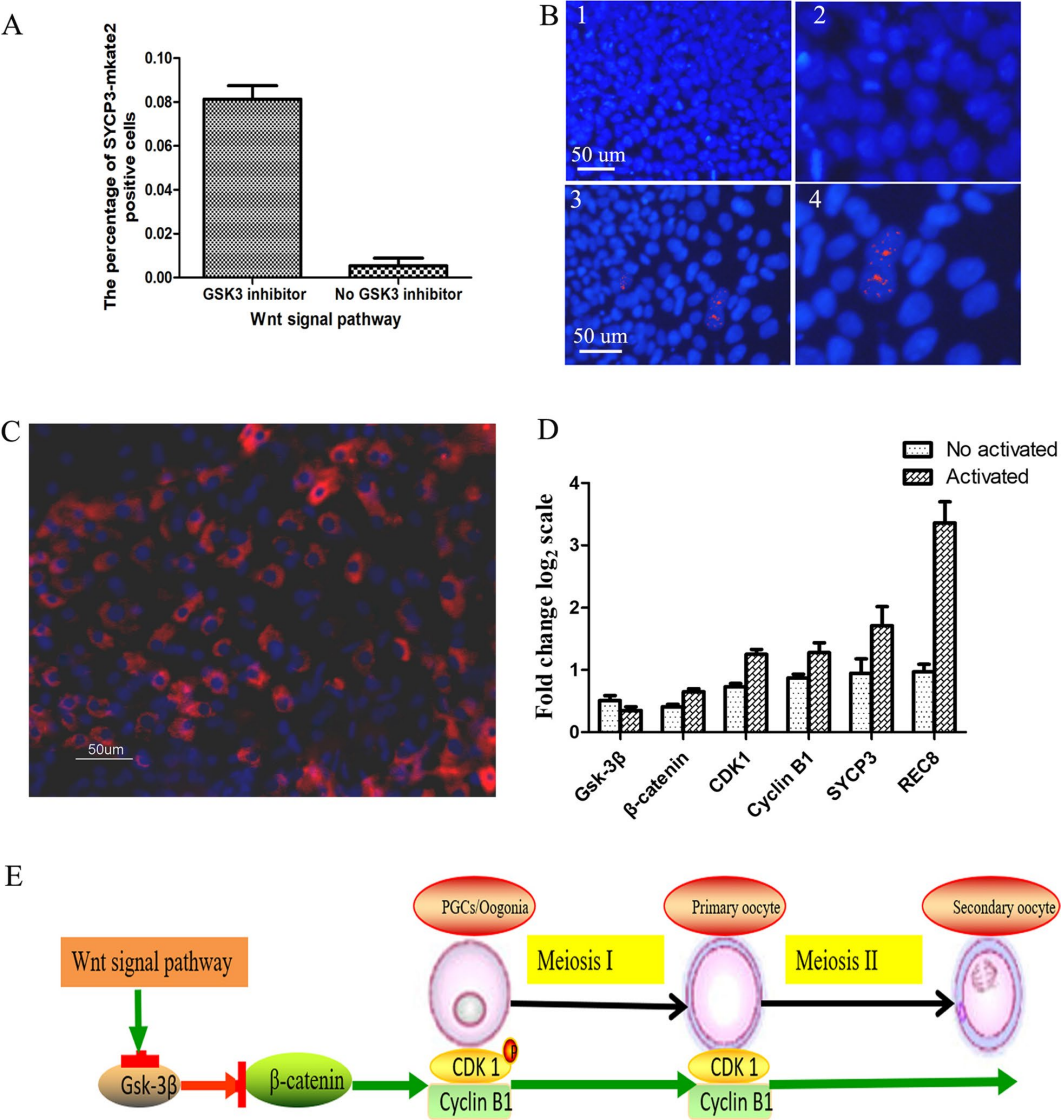
In vitro activation of dormant human PGCs/oogonia overcomes meiosis phase arrest through the Wnt signaling pathway. **A** Percentage of SYCP3-mkate2-positive cells among human PGCs/oogonia treated with the GSK3 inhibitor was 8.2 ± 1.32%, while few cells were mkate2-positive. **B** Activated PGCs aggregated with human fetal gonad somatic cells differentiated into SCP3-positive meiocytes, in contrast to the pseudoaggregates formed from only human gonad somatic cells that showed no SCP3 meiocytes and were used as a negative control. B-1: Negative control; B-2: magnifications of the negative control; B-3: positive control; and B-4: magnifications of the negative control. **C** Expression of *DDX4* in the ovary reconstituted with a mixture of human activated PGCs and fetal somatic cells. **D** Increased relative expression levels of SP3 and Dmc1 in PGCs induced from HEF-iPSCs. **D** Gene expression of *GSK-3β*, *β-catenin*, *CDK1*, *Cyclin B1*, *SYCP3* and *REC8* in the activated group and nonactivated group. **E** Proposed effect of the Wnt signaling pathway on meiotic resumption and progression in the differentiation of PGCs/oogonia

In our study, we explored whether the in vitro activation of dormant human PGCs after stimulation of the Wnt pathway caused them to resume meiosis. Human PGCs were cocultured with human fetal ovarian somatic cells of the abortus. We performed treated a mixture of human PGCs and fetal ovarian somatic cells with 3 mM GSK3 inhibitor CHIR99021 for 8 h in culture. Our results showed that the percentage of SYCP3-mkate2-positive cells among human PGCs treated with the GSK3 inhibitor was 8.2 ± 1.32%, while few cells were mkate2-positive, indicating the activation of dormant primordial follicles and increased folliculogenesis (Fig. 4A). Activated PGCs aggregated with human fetal gonad somatic cells were differentiated into SCP3-positive meiocytes, in contrast to the pseudoaggregates formed from only human gonad somatic cells that showed no SCP3 meiocytes and were used as a negative control (Fig. 4B). Immunofluorescence analysis of the reconstituted ovary showed that DDX4, which is a germ cell marker that begins to be expressed in gonadal PGCs, was positively expressed (Fig. 4C). To elucidate the possible molecular mechanism of meiotic resumption by the Wnt signaling pathway, we analyzed the gene expression of *GSK-3β*, *β-catenin*, *CDK1*, *Cyclin B1*, *SYCP3* and *REC8* (Fig. 4D and Additional file 4). The results suggest that regulation of *GSK-3β* expression is an important mechanism that controls the timing of meiotic resumption and that the Wnt signaling pathway likely also plays important roles in meiotic resumption. Based on these observations, we proposed an effect of the Wnt signaling pathway on meiotic resumption and progression in the differentiation of PGCs or oogonia into oocytes (Fig. 4E).

### Human activated PGC-derived oogonia display the status of meiotic recombination and resumed meiosis

**Fig. 5:**
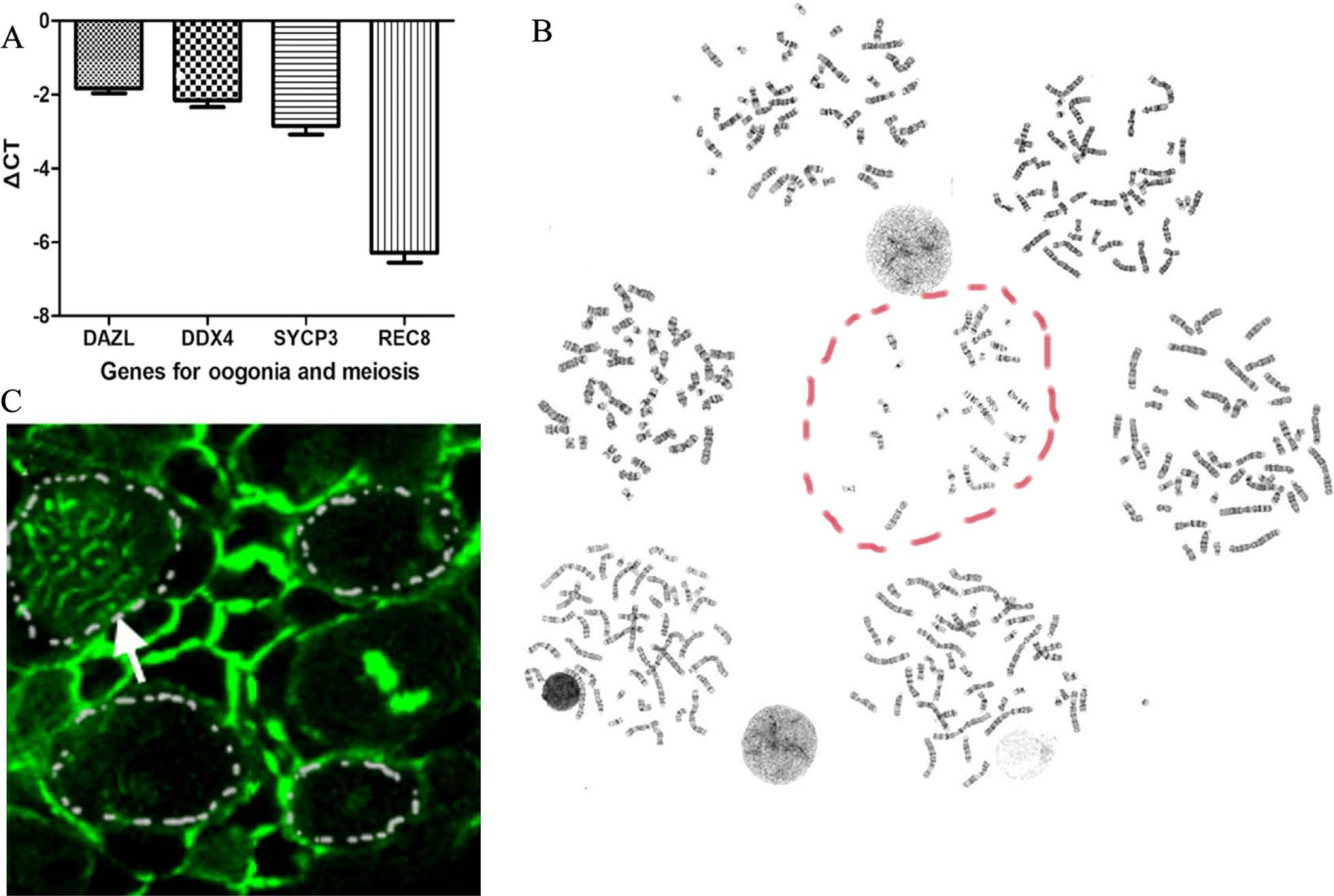
Human activated PGC-derived oogonia displayed a state of meiotic recombination and resumed meiosis. **A** Human activated PGC-derived cells upregulated genes for oogonia and gonocytes (*DAZL* and *DDX4*) and genes for meiosis (*SYCP3* and *REC8*). **B** In all 50 mitotic phases, 2 haploid karyotypes were found. **C** Chromosome pairing and crossover occurred in the mixture of human activated PGCs/oogonia and fetal somatic cells after transplantation into the ovarian nests of SCID mice

After in vitro activation, the treated mixture of human activated PGCs and fetal somatic cells was transplanted into the ovarian nests of SCID mice for folliculogenesis. We examined the expression of key genes using quantitative polymerase chain reaction. Human activated PGC-derived cells upregulate genes for oogonia and gonocytes (DAZL and DDX4) and meiosis (SYCP3 and REC8) (Fig. 5A and Table 1). This gene expression leads to the generation of oogonia, which are a state responding to the signal for meiotic entry and the preparation for meiotic recombination. Immunohistochemistry showed that chromosome pairing and crossover occurred (Fig. 5B), indicating the occurrence of meiosis. The karyotypes of the mixture of human activated PGCs and fetal somatic cells were analyzed. In all 50 mitotic phases, 2 haploid karyotypes were found (Fig. 5C), indicating the completion of meiosis. We then expanded the sample size. Five hundred mitotic phases were further analyzed, and 16 haploid karyotypes were found. The ratio of transformation from mitosis to meiosis was 3.2%. Based on these results, we confirmed that human activated PGC-derived oogonia displayed a state of meiotic recombination, resumed meiosis, and completed meiosis following in vitro activation and in vivo maturation.

**Table 1:** The primer sequences for the up-regulate genes for oogonia and gonocytes in this study are listed

Gene	Forward primer	Reverse primer
*DAZL*	TTTTTGTCTTTGTTGGAGTGAAGCA	ACAGTATCAGCAATAGGCAGAAGCA
*DDX4*	ACTGATACAAATGGTGTTAACTGGGA	AAACATGTCTAAGCCCCCTAAAGAA
*SYCP3*	CTTCCATGAAACAGCAGCAGCA	GGTTCAAGTTCTTTCTTCAAAGAGTCA
*REC8*	CCCTCTCCTCGCCTCTTGACCA	GAATCTGGGCCCCGGCTGGAT

## Discussion

Herein, human PGCs and oogonia were efficiently induced from iPSCs on feeder cells. Next, dormant PGCs or oogonia resumed meiosis and then differentiated into primary oocytes through the in vitro activation of the Wnt signaling pathway. Finally, we developed a new coculture system by reconstructing human ovarian nests in vitro using human embryonic ovarian somatic cells to facilitate the differentiation of oocytes. Our results show that the codifferentiated human activated PGCs in the reconstructed human ovarian nests resumed meiosis and differentiation into primary oocytes. These findings established a new strategy for germline competence in primary oocytes and provided a critical step toward human in vitro and in vivo gametogenesis.

The induction differentiation of human germ cells from human ESCs/iPSCs has been previously reported [27, 28]. However, there were several weaknesses in these studies. Inductive differentiation was often inefficient, the induced cells were poorly characterized, and it was difficult or impossible to monitor the differentiation process in human PGC specification. In our culture system, a higher efficiency of PGC induction from iPSCs was achieved in the feeder group.

Dormant human PGCs/oogonia resumed meiosis after stimulation of the Wnt pathway. The transformation from mitosis to meiosis is an important molecular event for successful in vitro gametogenesis from human PSCs. The mechanisms of meiosis resumption at the oogonium stage after differentiation into PGCs have not been clearly explained, although some studies have reported that dormant human PGCs/oogonia passed through the development bottleneck and entered the meiosis phase following stimulation of the AKT and mTOR signaling pathways [29]. Our results showed that the activation of the Wnt signaling pathway by regulating the expression of the *CDK1/CyclinB1* complex via *GSK-3β* could cause cells to resume meiosis. Future studies are underway and will lead to a better understanding of the molecular mechanisms of human oocyte development.

We developed a new coculture system by reconstructing ovarian nests in vitro using human fetal ovarian somatic cells from an abortus to facilitate the differentiation of oocytes. To explore the differentiation potential of human PGCs, allogeneic reconstituted ovaries were used to induce the differentiation of human PGCs by aggregation with mouse embryonic ovarian somatic cells [30]. Mouse embryonic ovarian somatic cells might not support such human germ cell differentiation processes, particularly under suboptimal culture conditions. The allogeneic reconstituted ovary system might not be an efficient culture system for the further differentiation of human PGCs/oogonia into primary oocytes with the progression of meiotic prophase I or for the subsequent formation of primordial/primary follicles [30]. Therefore, the conditions and molecular mechanism of the differentiation of human PGCs/oogonia into primary oocytes must be identified, requiring the use of human fetal ovarian somatic cells to form isogeneic reconstituted human ovaries. In our study, human fetal ovarian somatic cells from an abortus were used to form IROvaries to facilitate the differentiation of human PGCs/oogonia into oocytes. Such an endeavor will be key to achieving human gametogenesis in vitro and in vivo. Additionally, ovary organoids and 3D printing are other ways to explore the differentiation of oocytes from human iPSCs.

## Conclusion

Overall, we provided a robust approach for the stepwise differentiation of human iPSCs into oocytes in vitro, which involves the transformation from mitosis to meiosis. Our exploration could facilitate the generation of haploid human oocytes in vitro and in vivo with the goal of treating infertility.

## Supplementary Information

**Additional file 1.** The stable VASA-GFP-iPSCs possess totipotency of three germ layer differentiation. **A** All colonies stained positive Oct-4, Nanog, SSEA-4 and Sox2. **B** RT-PCR detection of pluripotency-related genes and differentiation-related genes in H9, iPSCs, VASA-GFP-iPSCs and hEF cells showed that pluripotency-related genes all expressed in H9, iPSCs, VASA-GFP-iPSCs, but not differentiation-related genes. **C** G-banding of VASA-GFP-iPSCs at passage 15 showed normal karyotype.

**Additional file 2.** The CD38, BLIMP1 and SOX17 protein levels were further identified by immunofluorescence. **A** The CD38 protein levels were further identified by immunofluorescence in culture system at Day 4 and Day 8. **B** The BLIMP1 protein levels were further identified by immunofluorescence in culture system at Day 4 and Day 8. **C** The SOX17 protein levels were further identified by immunofluorescence in culture system at Day 4 and Day 8.

**Additional file 3.** The expression of SYCP3 in SYCP3-mkate2 knock-in reporter iPSCs, iPSCs, Testis and hEF by PCR.

**Additional file 4.** The primer sequences for the genes examined in this study are listed.

## Data Availability

All data generated or analyzed during this study are included in this article.

## Abbreviations

PGCs: Primordial germ cells
iPSCs: Induced pluripotent stem cells
ESCs: Embryonic stem cells
EB: Embryonic body
EpiLCs: Epiblast-like cells
AMH: Anti-Mullerian hormone
MEFs: Mouse embryonic fibroblasts
SYCP3: Synaptonemal complex protein 3
H3K27me3: Histone H3 lysine 27 trimethylation
IrOvaries: Isogeneic reconstituted ovaries
TGF-β: Transforming growth factor-β
bFGF: Basic fibroblast growth factor

## References

[1] Rowe RG, Daley GQ (2019). Induced pluripotent stem cells in disease modelling and drug discovery. Nat Rev Genet.

[2] Dakhore S, Nayer B, Hasegawa K (2018). Human pluripotent stem cell culture: current status, challenges, and advancement. Stem Cells Int.

[3] Nakamura T, Okamoto I, Sasaki K, Yabuta Y, Iwatani C, Tsuchiya H, Seita Y, Nakamura S, Yamamoto T, Saitou M (2016). A developmental coordinate of pluripotency among mice, monkeys and humans. Nature.

[4] Hendriks S, Dancet EA, van Pelt AM, Hamer G, Repping S (2015). Artificial gametes: a systematic review of biological progress towards clinical application. Hum Reprod Update.

[5] Hübner K, Fuhrmann G, Christenson LK, Kehler J, Reinbold R, De La Fuente R, Wood J, Strauss JF, Boiani M, Schöler HR (2003). Derivation of oocytes from mouse embryonic stem cells. Science.

[6] Geijsen N, Horoschak M, Kim K, Gribnau J, Eggan K, Daley GQ (2004). Derivation of embryonic germ cells and male gametes from embryonic stem cells. Nature.

[7] Lacham-Kaplan O, Chy H, Trounson A (2006). Testicular cell conditioned medium supports differentiation of embryonic stem cells into ovarian structures containing oocytes. Stem Cells.

[8] Hayashi K, Ohta H, Kurimoto K, Aramaki S, Saitou M (2011). Reconstitution of the mouse germ cell specification pathway in culture by pluripotent stem cells. Cell.

[9] Hayashi K, Ogushi S, Kurimoto K, Shimamoto S, Ohta H, Saitou M (2012). Offspring from oocytes derived from in vitro primordial germ cell-like cells in mice. Science.

[10] Hayashi K, Saitou M (2013). Generation of eggs from mouse embryonic stem cells and induced pluripotent stem cells. Nat Protoc.

[11] Zhou Q, Wang M, Yuan Y, Wang X, Fu R, Wan H, Xie M, Liu M, Guo X, Zheng Y, Feng G, Shi Q, Zhao XY, Sha J, Zhou Q (2016). Complete meiosis from embryonic stem cell-derived germ cells in vitro. Cell Stem Cell.

[12] Sosa E, Chen D, Rojas EJ, Hennebold JD, Peters KA, Wu Z, Lam TN, Mitchell JM, Sukhwani M, Tailor RC, Meistrich ML, Orwig KE, Shetty G, Clark AT (2018). Differentiation of primate primordial germ cell-like cells following transplantation into the adult gonadal niche. Nat Commun.

[13] Richards M, Fong CY, Bongso A (2010). Comparative evaluation of different in vitro systems that stimulate germ cell differentiation in human embryonic stem cells. Fertil Steril.

[14] Lan CW, Chen MJ, Jan PS, Chen HF, Ho HN (2013). Differentiation of human embryonic stem cells into functional ovarian granulosa-like cells. J Clin Endocrinol Metab.

[15] Eguizabal C, Montserrat N, Vassena R, Barragan M, Garreta E, Garcia-Quevedo L, Vidal F, Giorgetti A, Veiga A, Izpisua Belmonte JC (2011). Complete meiosis from human induced pluripotent stem cells. Stem Cells.

[16] White YA, Woods DC, Takai Y, Ishihara O, Seki H, Tilly JL (2012). Oocyte formation by mitotically active germ cells purified from ovaries of reproductive-age women. Nat Med.

[17] Irie N, Weinberger L, Tang WW, Kobayashi T, Viukov S, Manor YS, Dietmann S, Hanna JH, Surani MA (2015). SOX17 is a critical specifier of human primordial germ cell fate. Cell.

[18] Yang S, Ding SF, Jiang XL, Sun BN, Xu QH (2016). Establishment and adipocyte differentiation of polycystic ovary syndrome-derived induced pluripotent stem cells. Cell Prolif.

[19] Sasaki K, Yokobayashi S, Nakamura T, Okamoto I, Yabuta Y, Kurimoto K, Ohta H, Moritoki Y, Iwatani C, Tsuchiya H, Nakamura S, Sekiguchi K, Sakuma T, Yamamoto T, Mori T, Woltjen K, Nakagawa M, Yamamoto T, Takahashi K, Yamanaka S, Saitou M (2015). Robust in vitro induction of human germ cell fate from pluripotent stem cells. Cell Stem Cell.

[20] Yang S, Ding S, He S, He L, Gao K, Peng S, Shuai C (2019). Differentiation of primordial germ cells from premature ovarian insufficiency-derived induced pluripotent stem cells. Stem Cell Res Ther.

[21] Livak SN, Schmittgen TD (2001). Analysis of relative gene expression data using real-time quantitative PCR and the 2-△△ct methods. Methods.

[22] Herbert M, Levasseur M, Homer H, Yallop K, Murdoch A, McDougall A (2003). Homologue disjunction in mouse oocytes requires proteolysis of securin and cyclin B1. Nat Cell Biol.

[23] Yu J, Raia P, Ghent CM, Raisch T, Sadian Y, Cavadini S, Sabale PM, Barford D, Raunser S, Morgan DO, Boland A (2021). Structural basis of human separase regulation by securin and CDK1-cyclin B1. Nature.

[24] Li J, Qian WP, Sun QY (2019). Cyclins regulating oocyte meiotic cell cycle progression. Biol Reprod.

[25] Gutierrez GJ, Vögtlin A, Castro A, Ferby I, Salvagiotto G, Ronai Z, Lorca T, Nebreda AR (2006). Meiotic regulation of the CDK activator RINGO/Speedy by ubiquitin-proteasome-mediated processing and degradation. Nat Cell Biol.

[26] Adhikari D, Liu K (2014). The regulation of maturation promoting factor during prophase I arrest and meiotic entry in mammalian oocytes. Mol Cell Endocrinol.

[27] Yamashiro C, Sasaki K, Yabuta Y, Kojima Y, Nakamura T, Okamoto I, Yokobayashi S, Murase Y, Ishikura Y, Shirane K, Sasaki H, Yamamoto T, Saitou M (2018). Generation of human oogonia from induced pluripotent stem cells in vitro. Science.

[28] Yamashiro C, Sasaki K, Yokobayashi S, Kojima Y, Saitou M (2020). Generation of human oogonia from induced pluripotent stem cells in culture. Nat Protoc.

[29] Li J, Kawamura K, Cheng Y, Liu S, Klein C, Liu S, Duan EK, Hsueh AJ (2010). Activation of dormant ovarian follicles to generate mature eggs. Proc Natl Acad Sci USA.

[30] Yamashiro C, Sasaki K, Yokobayashi S, Kojima Y, Saitou M (2018). Generation of human oogonia from induced pluripotent stem cells in vitro. Science.

