# Supplementary figures and images for: Meiosis resumption in human primordial germ cells from induced pluripotent stem cells by in vitro activation and reconstruction of ovarian nests
Source: Stem Cell Res Ther. 2022 Jul 26;13:339. doi: 10.1186/s13287-022-03019-3 (PMC9327357; doi:10.1186/s13287-022-03019-3)

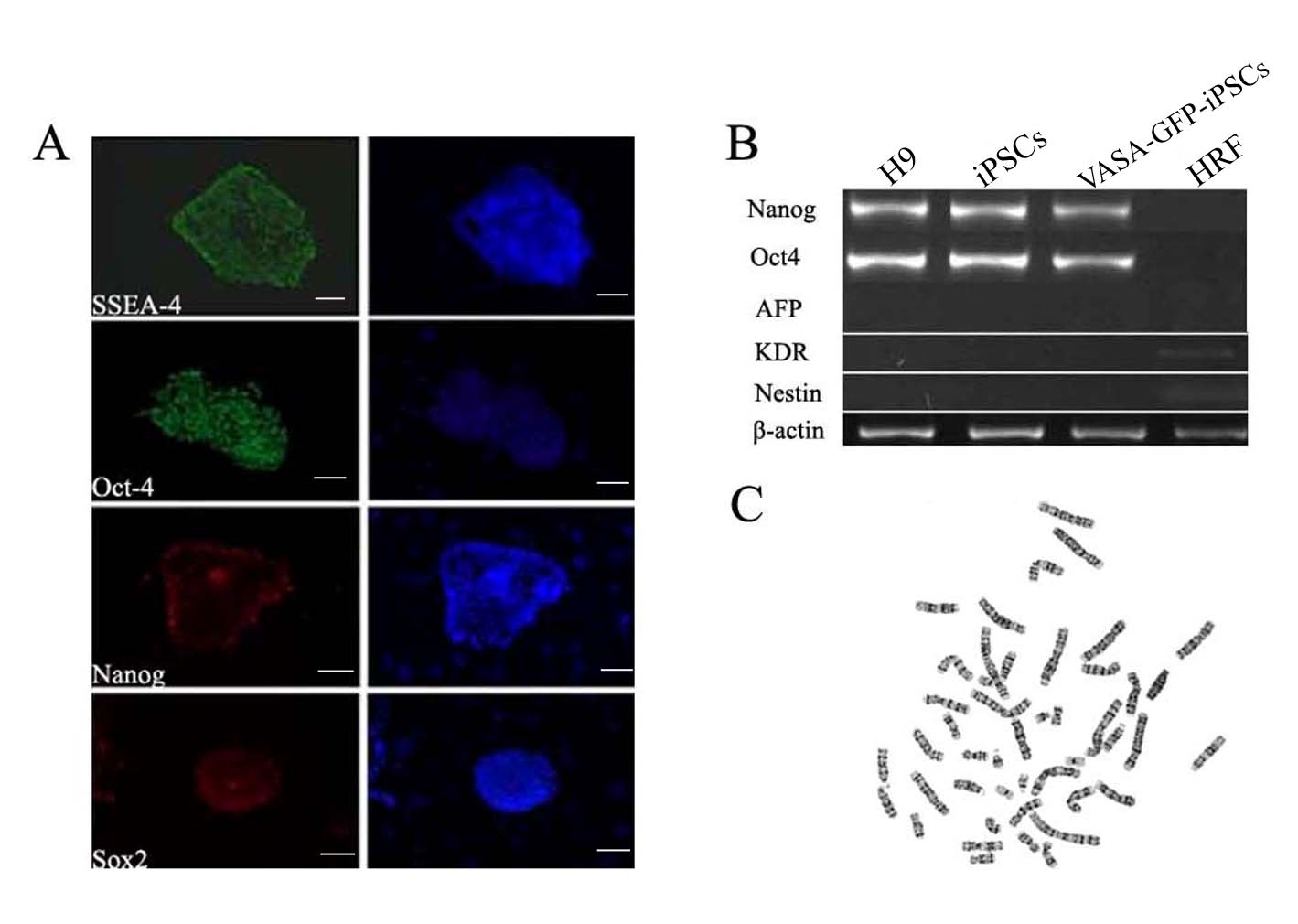

Supplement: Supplementary file 1 — Additional file 1. The stable VASA-GFP-iPSCs possess totipotency of three germ layer differentiation. A All colonies stained positive Oct-4, Nanog, SSEA-4 and Sox2. B RT-PCR detection of pluripotency-related genes and differentiation-related genes in H9, iPSCs, VASA-GFP-iPSCs and hEF cells showed that pluripotency-related genes all expressed in H9, iPSCs, VASA-GFP-iPSCs, but not differentiation-related genes. C G-banding of VASA-GFP-iPSCs at passage 15 showed normal karyotype. [file 13287_2022_3019_MOESM1_ESM.jpg]

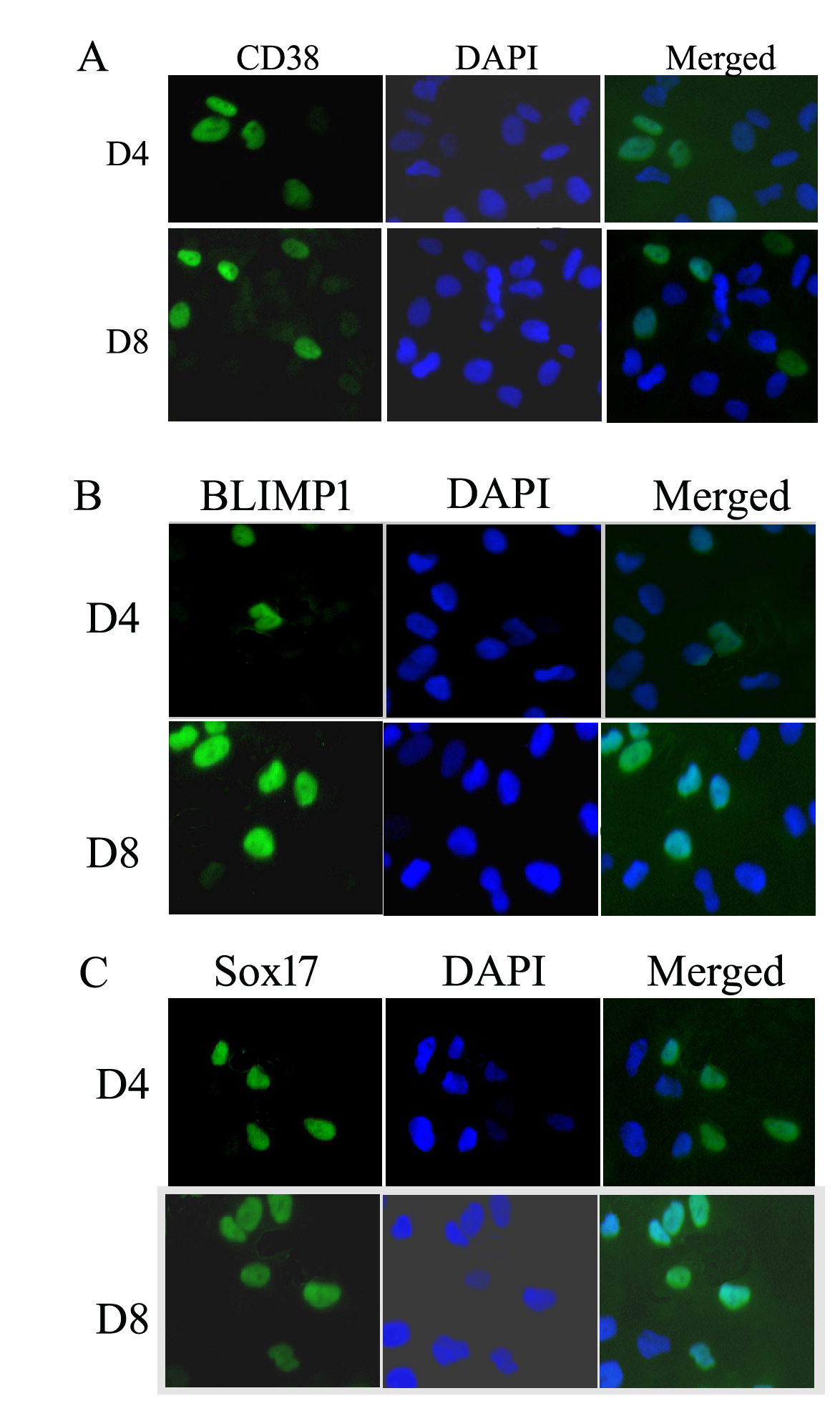

Supplement: Supplementary file 2 — Additional file 2. The CD38, BLIMP1 and SOX17 protein levels were further identified by immunofluorescence. A The CD38 protein levels were further identified by immunofluorescence in culture system at Day 4 and Day 8. B The BLIMP1 protein levels were further identified by immunofluorescence in culture system at Day 4 and Day 8. C The SOX17 protein levels were further identified by immunofluorescence in culture system at Day 4 and Day 8. [file 13287_2022_3019_MOESM2_ESM.jpg]

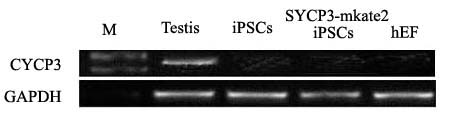

Supplement: Supplementary file 3 — Additional file 3. The expression of SYCP3 in SYCP3-mkate2 knock-in reporter iPSCs, iPSCs, Testis and hEF by PCR. [file 13287_2022_3019_MOESM3_ESM.jpg]
