# Supplementary material for: Meiosis resumption in human primordial germ cells from induced pluripotent stem cells by in vitro activation and reconstruction of ovarian nests
Source: Stem Cell Res Ther. 2022 Jul 26;13:339. doi: 10.1186/s13287-022-03019-3 (PMC9327357; doi:10.1186/s13287-022-03019-3)
Supplement: Supplementary file 4 — Additional file 4. The primer sequences for the genes examined in this study are listed. [file 13287_2022_3019_MOESM4_ESM.docx]

Table 1 The primer sequences for the genes examined in this study are listed

| gene | Forward primer | Reverse primer |  |
| --- | --- | --- | --- |
| *Gsk-3β* | TTTTTGTCTTTGTTGGAGTGAAGCA | ACAGTATCAGCAATAGGCAGAAGCA | |
| *β-catenin* | ACTGATACAAATGGTGTTAACTGGGA | AAACATGTCTAAGCCCCCTAAAGAA | |
| *CDK1* | CTTCCATGAAACAGCAGCAGCA | GGTTCAAGTTCTTTCTTCAAAGAGTCA | |
| *Cyclin B1* | CCCTCTCCTCGCCTCTTGACCA | GAATCTGGGCCCCGGCTGGAT | |
| *SYCP3* | CTTCCATGAAACAGCAGCAGCA | GGTTCAAGTTCTTTCTTCAAAGAGTCA | |
| *ERC8* | CCCTCTCCTCGCCTCTTGACCA | GAATCTGGGCCCCGGCTGGAT | |
